# Yeast Heterologous Expression Systems for the Study of Plant Membrane Proteins

**DOI:** 10.3390/ijms241310768

**Published:** 2023-06-28

**Authors:** Larissa G. Popova, Dmitrii E. Khramov, Olga I. Nedelyaeva, Vadim S. Volkov

**Affiliations:** K.A. Timiryazev Institute of Plant Physiology RAS, 127276 Moscow, Russia; khramov.de@yandex.ru (D.E.K.); olga.nedelyaeva@yandex.ru (O.I.N.)

**Keywords:** baker’s yeasts, heterologous expression, methylotrophic yeasts, plant membrane proteins, recombinant proteins, *Pichia pastoris*, *Saccharomyces cerevisiae*

## Abstract

Researchers are often interested in proteins that are present in cells in small ratios compared to the total amount of proteins. These proteins include transcription factors, hormones and specific membrane proteins. However, sufficient amounts of well-purified protein preparations are required for functional and structural studies of these proteins, including the creation of artificial proteoliposomes and the growth of protein 2D and 3D crystals. This aim can be achieved by the expression of the target protein in a heterologous system. This review describes the applications of yeast heterologous expression systems in studies of plant membrane proteins. An initial brief description introduces the widely used heterologous expression systems of the baker’s yeast *Saccharomyces cerevisiae* and the methylotrophic yeast *Pichia pastoris*. *S. cerevisiae* is further considered a convenient model system for functional studies of heterologously expressed proteins, while *P. pastoris* has the advantage of using these yeast cells as factories for producing large quantities of proteins of interest. The application of both expression systems is described for functional and structural studies of membrane proteins from plants, namely, K^+^- and Na^+^-transporters, various ATPases and anion transporters, and other transport proteins.

## 1. Introduction

Research on the structure and function of proteins often requires heterologous expression of the proteins. It is especially important for membrane proteins, which are localised within or linked to biological membranes. Typically, the content of individual transporters is low in biological membranes, so with the background of the other membrane proteins, isolation of sufficient quantities of the required protein for structural and/or functional studies is difficult. However, the importance of functional and structural studies of membrane proteins is obvious, since 20–30% of all genes, both in prokaryotes and eukaryotes, encode membrane proteins that often form supramolecular complexes and are involved in various vital processes [1,2].

At present, several types of prokaryotic and eukaryotic systems are used for the heterologous expression of membrane proteins. The expression of prokaryotic and eukaryotic hydrophilic soluble proteins has often been successful in the Gram-negative bacterium *Escherichia coli* [3]. However, this prokaryotic expression system did not prove to be so fruitful for eukaryotic hydrophobic membrane proteins. This is largely due to the absence in the prokaryotic cell of an effective system targeting eukaryotic membrane proteins to the plasma membrane, as well as the absence of protein factors required for proper folding and posttranslational modifications of heterologously expressed eukaryotic membrane proteins [4]. In addition, many membrane proteins seem to be toxic when expressed in bacteria [5]. Still, there are examples when membrane proteins from eukaryotes have been expressed and targeted to prokaryotic membranes. For example, the functional expression of the human KDEL receptor and transporters belonging to the ABC transporter family and yeast transporters belonging to the mitochondrial carrier family have been described in the Gram-positive bacterium *Lactococcus lactis* (reviewed in [6]). Examples of successful heterologous expression of plant membrane proteins in *E. coli* cells include the expression of inward-rectifying potassium channels KAT1 and AKT from the model plant *Arabidopsis thaliana* [7] and the expression of the two-pore K^+^ channel (NtTPK1) from *Nicotiana tabacum* [8]. In both reports, heterologous plant K^+^ transporters functionally complemented *E. coli* strains deficient in K^+^ uptake systems.

Eukaryotic expression systems are required in most cases to express eukaryotic membrane proteins heterologously. At present, several expression systems are widely used, including expression in yeast cells, *Xenopus* oocytes, insect cells (baculovirus/insect cell system), mammalian cells and plant cells (reviewed in [9]). For example, the baculoviral expression system based on insect cells infected with recombinant baculovirus provides good expression results. Recombinant proteins in insect cells are often active and appear to undergo a series of post-translational modifications necessary to produce recombinant proteins that are very similar to authentic proteins. In this baculovirus/insect cell system, high-purity preparations for several GPCR proteins (G-protein coupled receptors) were obtained, which were further used for structural studies [10]. Mammalian cell lines (HEK-293, COS-1) were also reliable hosts for heterologous expression with subsequent crystallisation of, e.g., a few rhodopsins (reviewed in [9]).

The signal transduction pathways, mechanisms of transcription and translation are conserved among representatives of eukaryotic kingdoms, explaining the advantages of using eukaryotic systems for heterologous expression of eukaryotic proteins. Moreover, compatible mechanisms of posttranslational protein modifications (glycosylation, processing of signal sequences, and formation of disulfide bonds) that ensure correct protein folding and their translocation to the required cellular compartments are all functional in different eukaryotic systems. However, the disadvantages of insect and animal cell lines for heterologous expression include complicated tissue culture methodologies, higher costs and much longer steps for the isolation of required proteins in comparison to prokaryotic expression systems [11].

Reliable results can be achieved using yeast systems of heterologous expressions. These systems possess the advantages of low cost, and they are amenable to genetic manipulations, biologically safe, provide sufficient amounts of cell biomass when using simple growth media, and hence, provide an opportunity to produce large amounts of eukaryotic integral membrane proteins [12]. Various yeast systems are available for the heterologous expression of eukaryotic proteins. *Saccharomyces cerevisiae* and *Kluyveromyces lactis* (able to utilize lactose), *Yarrowia lipolytica* (utilizes hydrocarbons, fatty acids and lipids) and methylotrophic species *Candida boidinii, Pichia pastoris*, *P. methanolica* and *Hansenula polymorpha* are widely used for genetic studies [12]. However, the most popular for heterologous protein expression are the two species, baker’s yeast *S. cerevisiae* and methylotrophic yeast *P. pastoris*, expression in which is a well-established and widely used technique. The identification of many plant transporters has been achieved through the use of heterologous expression in these types of yeast. Experimental systems using *S. cerevisiae* and *P. pastoris* are considered further in this review, particularly for proteins involved in ion transport in plants. Approximately 5–10% of the entire plant proteome represents membrane transporters [13]. These proteins play important roles in diverse aspects of plant cell biology and physiology, including nutrient uptake, cell signalling, energy transduction and ionic homeostasis. Therefore, insights into these processes require a detailed understanding of the molecular mechanisms and regulatory controls that determine the activity of the transporters. Thus, the cloning of ion transporters and the use of heterologous expression systems in which isolated transporters are functionally studied are of particular importance.

## 2. Baker’s Yeast *Saccharomyces cerevisiae* for Functional Study of Plant Membrane Proteins

Baker’s yeast *S. cerevisiae* was the first eukaryotic system used for heterologous expression. Yeast genetics and physiology are well characterized, and this yeast remains the most helpful tool to elucidate the functions of proteins of different origins [14]. The convenience of this model system for studying, in particular, plant proteins is explained not only by the multiple simple and available routine methods of yeast manipulation but also by the fact that yeasts possess a eukaryotic system of posttranslational modifications of synthesised proteins. At present, there exists a large collection of *S. cerevisiae* mutants that are used for functional studies of heterologously expressed proteins (http://www.yeastgenome.org (accessed on 25 June 2023); [9]). The cells of *S. cerevisiae* successfully served as vehicles for the expression of many proteins of plant origin, including membrane proteins such as phosphate transporters AtPT1 и AtPT2 [15], Ca^2+^-ATPase of endoplasmic reticulum ECA1 [16], nitrate transporter NRT1.1 [17], peptide transporter of plasma membrane AtPTR1 [18] (all of these proteins are from the model plant *A. thaliana*), sulphate transporters LeST1-1 and LeST1-2 from tomato *Lycopersicon esculentum* [19] and Na^+^-ATPase PpENA1 from moss *Physcomitrella patens* [20]. To identify stress-tolerance-related plant genes the functional screening of the genes in yeast can be applied. For example, for the halophyte *Ipomea pes-caprae*, large-scale screening of genes involved in the salt stress response was performed via a full-length cDNA over-expressing gene hunting system (FOX hunting system, the gain-of-function system [21]) with a functional screening of a cDNA library using a salt-sensitive yeast mutant strain [22].

### 2.1. Vector Systems Used for Heterologous Expression in Cells of S. cerevisiae

**Figure 1:**
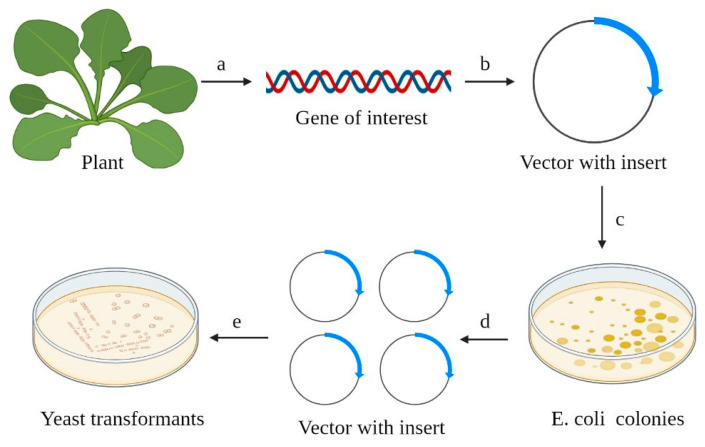
The key steps in the expression of heterologous protein in yeast cells. (a) The gene of interest (cDNA) is isolated from a plant sample; (b) the cDNA (blue arrow) is integrated into the host vector; (c,d) *E. coli* is routinely used for construct amplification; (e) yeast cells are transformed by the vector containing the cDNA of gene of interest. Selection of colonies (*E. coli*, yeast colonies) is carried out on selective media.

Figure 1 shows the scheme of an experiment on cloning the gene of interest from plants in yeast cells. All types of plasmids used for the design of constructions of heterologous expression in yeast cells are binary (or shuttle) vectors [12,23]. They contain the sequences derived from bacterial plasmids and the components of a yeast replication system, permitting them to replicate in both *E. coli* cells and yeast cells. The ability to replicate in *E. coli* cells ensures the simplicity of manipulations and the convenience to obtain large amounts of plasmids.

**Figure 2:**
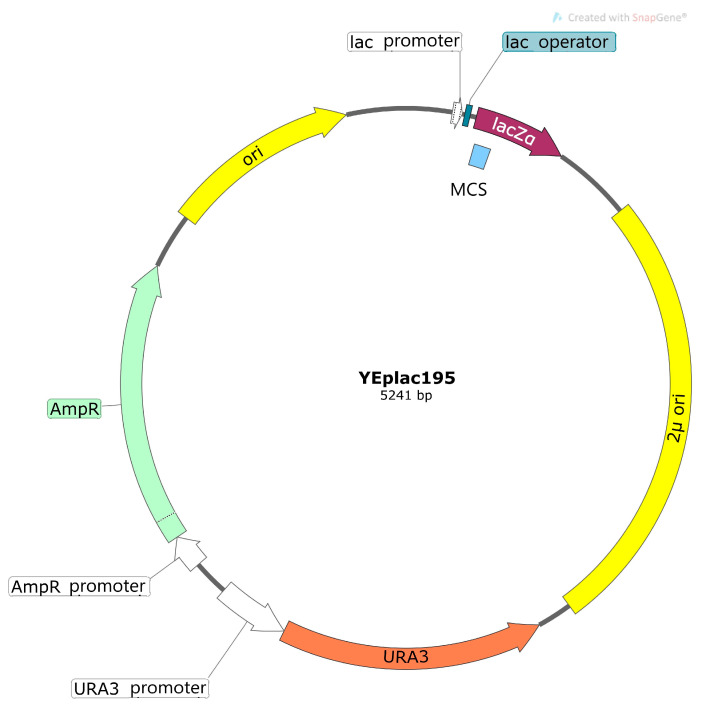
Scheme of yeast episome plasmid. MCS—multiple cloning site (polylinker); ori—bacterial replication site; AmpR—gene of resistance to ampicillin (selective markers for the selection of bacterial transformants); 2µ ori—ori site of yeast 2µ-plasmid; URA3—gene of orotidine-5′phosphate decarboxylase (selective markers for the selection of yeast transformants).

The bacterial segment of the plasmid has elements that are required for plasmid replication in *E. coli* cells: bacterial ori (replication initiation site) and the selective gene of antibiotic resistance (for example, resistance to ampicillin) (Figure 2). Multiple unique restriction sites located in the region of the plasmid named polylinker allow the insertion of a heterologous coding sequence. The yeast part of the plasmid, in addition to the elements necessary for replication in the yeast cell (ori, ARS) or integration into its genome, contains additional selective markers for the selection of yeast transformants on selective media. For example, genes of β-isopropylmalate dehydrogenase (*LEU2*) or orotidine-5′phosphate decarboxylase (*URA3*) are often used for this purpose. Yeast strains deficient in the *LEU2* gene or *URA3* gene and subsequently auxotrophic in the components of growth medium (leucine or uracil, correspondingly) are used for transformation. It allows the selection of yeast transformants in media without leucine or, correspondingly, uracil.

Based on the manner of replication in the yeast cells, the genetic constructions for the expression of heterologous proteins in *S. cerevisiae* can be classified into four groups [12]: YIp (yeast integrating plasmids), YRp (yeast replicative plasmids), YCp (yeast centromere plasmids) and YEp (Yeast Episome plasmids).

Integrative plasmids YIp do not contain yeast ori sites and are replicated when integrated into the yeast genome. They contain regions of homology with yeast DNA, and the integration into the genome is realised by homologous recombination. The vectors are stable, but typically the number of their copies in the genome is relatively low, which determines the low level of heterologous protein expression. This problem can be solved by integrating vectors into the region of tandem repeats of the rRNA gene. This integration allows for many copies of the cloned DNA sequences. In addition, the integration site can profoundly affect the levels of exogenous gene expression [24]. Integrative vectors find their applications in metabolic engineering in yeast, which often require stable integration of heterologous genes [25,26].

The replicative plasmids YRp, in addition to the vector sequence of YIp type, contain the yeast chromosome replication initiation site ARS (autonomously replicating sequence). Plasmids of the YRp type can autonomously replicate in yeast cells without integration into the chromosomes. However, after yeast cell divisions, YRp plasmids are distributed unevenly in daughter cells, and the progeny cells may lose the plasmids.

The constructions of YEp-type plasmids are based on episomal vectors that differ from integrative plasmids by the presence of the ori site of the yeast 2μ-plasmid (Figure 2). Their replication is independent of chromosome DNA; they are present in cells with large copy numbers (30 copies or more). The plasmids distribute unevenly in the progeny cells, but they are supported consistently due to the large copy number. Plasmids of the YEp type are used for achieving high expression levels of heterologous proteins. In many cases, the amount of the expressed protein is the most important factor, and these high-copy vectors are routinely used for the overexpression of recombinant proteins.

The vectors of the YCp type contain a yeast centromere sequence (CEN) fused to ARS. The fused sequence ARS/CEN is responsible for the replication and segregation of the YCp-type vectors. The vectors, as YEp-type vectors, replicate independently of chromosome DNA; they are stable and evenly distributed between the daughter cells, but they are represented by a small number of copies.

To express a heterologous protein, the coding part of its gene is integrated between the promoter of the yeast gene with high expression and its terminator [12]. One of the advantages of yeast systems is the presence of strong constitutive promoters. In *S. cerevisiae*, these promoters control the expression of proteins such as plasma membrane H^+^-ATPase (*PMA1*), glyceraldehyde-3-phosphate hydrogenase (*GPD*), phosphoglycerate kinase-1 (*PGK1*), alcohol dehydrogenase-1 (*ADH1*), and the transporter responsible for pleiotropic drug resistance (*PDR5*) [27]. Apart from the constitutive promoters, there are inducible promoters that can ensure the expression of heterologous proteins at a required time. This is especially important when expressing proteins that are toxic to cells. The set of inducible promoters includes promoters of galactose metabolism *GAL1-10* (induced by galactose), *PHO5* (induced by low concentrations of inorganic phosphate in an external medium) and *HSE* elements (induced by an increase in the temperature to 37 °C). A description of yeast strains used in laboratory practice, expression vectors, selection markers and promoters for recombinant protein production in various yeast strains is reviewed in [28]. 

### 2.2. Functional Complementation of Yeast Saccharomyces cerevisiae as a Method to Study Plant Potassium and Sodium Channels and Transporters

Most processes controlling ion homeostasis are essentially similar for cells of higher plants and *S. cerevisiae* [29]; therefore, the cells of *S. cerevisiae* are suitable as a model system to study ion transporters of plant origin. Some of the most successful examples of exploiting *S. cerevisiae* for heterologous expression of plant membrane proteins and their functional complementation are the studies of proteins that transport potassium and sodium.

There are several general experimental approaches to identify the genes of potassium and sodium transporters, determine the kinetic properties of the transporters and find their potential regulators employing *S. cerevisiae*: functional complementation using yeast mutants, high-throughput protein–protein interaction assay, the reconstitution of functional transport systems and the identification of plant genes capable of conferring salt tolerance upon overexpression (reviewed in [30]). One of the most widely used approaches to functionally characterise the transporters of K^+^ and Na^+^ is the functional complementation of yeast *S. cerevisiae* mutants that are defective in their own genes for the corresponding transporters.

**Figure 3:**
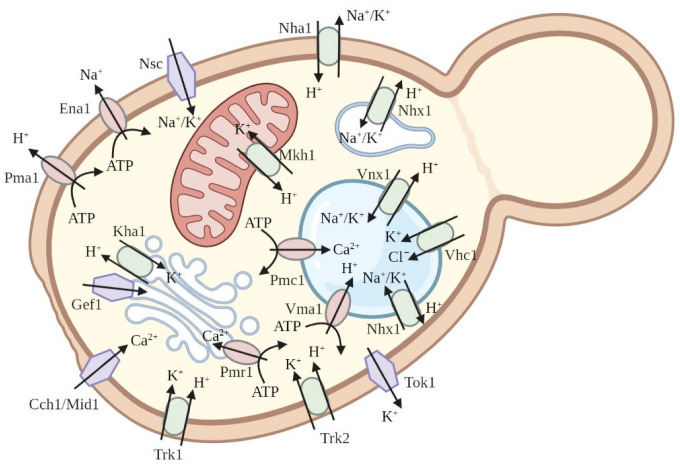
The major known plasma membrane and intracellular membrane ion transporters in the yeast cell are mentioned in the text.

Baker’s yeast *S. cerevisiae* possesses its own systems for potassium and sodium transport (Figure 3). The uptake of potassium from the external medium by the cells of *S. cerevisiae* depends mostly on two transporters in the yeast plasma membrane, Trk1 and Trk2 [31,32,33]. These integral membrane proteins ensure high-affinity potassium transport at the expense of the electrochemical gradient of H^+^ that is created by the yeast plasma membrane H^+^-ATPase Pma1 [34]. The yeast double mutant *trk1 trk2* is not able to grow in media with low concentrations of K^+^ (below 1 mM), but the growth of the double mutant is restored to the rate of the wild-type yeast when millimolar concentrations of K^+^ are added to the medium. Therefore, the double mutant *trk1 trk2* could be used for functional complementation when the heterologous expression of potassium transporters from other organisms in the mutant restores its growth under low K^+^ concentrations in the medium.

Under conditions of salt stress with high external Na^+^ concentration, Na^+^ enters the yeast cell via nonselective cation transporters in the plasma membrane (Nsc) and also via the potassium transporters Trk1 and Trk2 [33,35]. Then, two main systems function to expel Na^+^ from the cytoplasm of yeast cells. The first system is the P-type Na^+^-ATPase Ena1 [36,37], which is located in the plasma membrane. Ena1 actively pumps Na^+^ out of the cytoplasm and hydrolyses ATP to provide energy for this process. The genomes of many yeasts possess three to five tandem copies of the *ENA* gene; *ENA1* expression is induced upon exposure to salt and alkaline stresses [38]. When the external pH of yeast cells is low, the other system of Na^+^ export from the cytoplasm plays an important role, which is represented by the integral membrane protein Nha1 [39,40]. The Nha1 transporter functions as a cation/H^+^ antiporter with presumably similar selectivities for Na^+^ and K^+^ (reviewed in [41]). Strains lacking the *ENA* cluster and *NHA1* gene are highly salt sensitive [36,42]. Such strains are used to characterize plant genes involved in cation extrusion and salt tolerance.

The cells of *S. cerevisiae* are also equipped with additional transporters of potassium and sodium located at the vacuolar membrane. This group of transporters includes two proteins, Vnx1 and Vhc1. Protein Vnx1 is a cation/H^+^ antiporter that transports potassium or sodium to the vacuole [43]. Therefore, it participates in the detoxification of sodium via vacuolar compartmentalisation. The other protein of vacuolar membranes, Vhc1, is a K^+^/Cl^−^ symporter that takes part in maintaining the intracellular concentration of potassium and the morphology of the vacuole [44].

Other yeast cell compartments also have several ion transport proteins with selectivity for potassium and sodium. For example, the cation/H^+^ antiporter Nhx1 with high affinity to Na^+^ and K^+^ is located in the tonoplast and endosomal membranes; K^+^/H^+^- antiporter Kha1 functions in the Golgi apparatus; protein Mkh1, which exchanges H^+^ for K^+^, was detected in mitochondria [45].

The identification of plant genes involved in K^+^ uptake (K^+^ channels and transporters) is based mainly on complementation analysis of yeast mutants lacking *TRK1* and *TRK2* genes. The first plant potassium channels whose functions were proven by functional complementation of yeast *trk1 trk2* mutants (without their own high-affinity K^+^-transporters) were the proteins KAT1 [46] and AKT1 [47] from *A. thaliana*. Both proteins are inward-rectifying potassium channels belonging to the voltage-gated Shaker-type ion channel family; both are integral proteins of the plant cell plasma membrane, but they have different patterns of expression. KAT1 is expressed mainly in stomatal guard cells, where it takes part in the stomatal opening, whereas AKT1 is expressed in roots and is important for potassium uptake from soil [48]. Under heterologous expression in *S. cerevisiae*, each of the proteins was able to rescue the phenotype (restore the growth) of the double mutant *trk1 trk2* in a medium with low potassium [46,47]. Isoforms of KAT1 from other plants have also been identified (reviewed in [30]). For example, using a *trk1 trk2* mutant, a KAT1 homologue from rice (*Oriza sativa*) was characterized. The heterologous expression of OsKAT1 suppressed the K^+^-transport defective phenotype of *trk1 trk2*, suggesting the enhancement of K^+^ uptake by OsKAT1 [49].

Later, the approach with double mutant *trk1 trk2* was applied for the functional studies of other transporters, including plant high-affinity potassium transporters of the HKT family (high-affinity K^+^ Transporter), which transport K^+^ as well as Na^+^ in plants [50]. The phylogenetic and functional analyses of proteins belonging to the HKT family of transporters divide them into two groups, and the analyses added many details for their selectivity and functions [51,52]. Group I of HKT transporters includes transporters that mostly transport Na^+^ via the plasma membrane; they are involved in the recirculation of the ion via vascular tissues, whereas the HKT transporters of group II function as Na^+^-K^+^-symporters or K^+^-selective uniporters [52,53]. To date, the HKT transporters of group II have only been identified in monocotyledonous plants [51]. Higher sodium selectivity for HKT from group I is linked to a conserved serine residue forming a motif of S-G-G-G in the first pore loop, whereas the ability to transport K^+^ (and also Na^+^, depending on concentrations) for group II HKT transporters is associated with a glycine residue at the position (G-G-G-G motif) [52,53]. HKT transporters are of special interest since several members of this family play important roles in the salinity tolerance of plants [54,55]. The *Arabidopsis* AtHKT1 transporter has been shown to be a strong determinant of the salt tolerance of this species; it was demonstrated that AtHKT1 for Arabidopsis is located mainly in the phloem and also at the plasma membrane of xylem parenchyma cells [56,57]. Therefore, HKT’s role in the salinity tolerance of plants could be explained by the uptake of Na^+^ from xylem vessels, recirculation of Na^+^ via the phloem, and for glycophytes, prevention of accumulation of the toxic cation in photosynthetic tissues [56,57,58].

The first functionally characterised HKT transporter was HKT1 from wheat (*Triticum aestivum*; the protein TaHKT2;1 in modern classification); this protein was the first Na^+^-coupled K^+^-transporter described in higher plants [50,59]. HKT1 demonstrated properties of high-affinity Na^+^-K^+^-symporter when heterologously expressed in yeast *trk1 trk2* double mutant [60]. For similar concentrations of potassium and sodium, HKT1 transports/symports one Na^+^ with K^+^; for higher (millimolar) concentrations of sodium, Na^+^ ions can compete with K^+^ and substitute K^+^ in the cation binding sites of the transporter [59,60]. Therefore, at toxic (millimolar) levels of Na^+^, HKT1 mediates low-affinity Na^+^ uptake, whereas K^+^ uptake via the transporter is blocked [59]. Later, AtHKT1;1, the *Arabidopsis* protein homologue of the wheat HKT1 (TaHKT2;1) was identified; when expressed in heterologous systems such as *S. cerevisiae* (and *Xenopus laevis* oocytes), AtHKT1;1 shows a strong preference for Na^+^ selective transport [61].

Recently, a novel HKT gene, *SeHKT1;2*, was isolated and characterised from the halophyte *Salicornia europaea* [62]. The protein SeHKT1;2 belongs to subfamily I of HKT and shows high homology with other halophyte HKT proteins. *SeHKT1;2* expressed in the yeast *trk1 trk2* double mutant could not rescue the K^+^ uptake-defective phenotype of this strain. However, functional characterisation of SeHKT1;2 in yeast strains with disrupted *ENA1-4* genes encoding the isoforms of yeast Na^+^ exporting ATPase revealed that SeHKT1;2 contributes to facilitating Na^+^ uptake in this Na^+^-sensitive yeast strain, demonstrating that SeHKT1;2 selectively transports Na^+^ rather than K^+^.

In the *S. cerevisiae* mutant *trk1 trk2*, the low-affinity cation transporter (LCT1) from wheat was cloned and functionally characterised [63]. *LCT1* is expressed in low abundance in wheat roots and leaves. The expression of *LCT1* in *S. cerevisiae* demonstrated that the transporter LCT1 mediated low-affinity uptake of Na^+^ and Rb^+^ and could function as a component of multiple low-affinity Na^+^ and K^+^ uptake pathways in wheat roots. Further experiments have shown that (1) LCT1 complemented a yeast disruption mutant in the *MID1* gene, a non-*LCT1*-homologous yeast gene encoding a protein required for Ca^2+^ influx and mating [64], and (2) LCT1 mediates the high-affinity uptake of Ca^2+^ and Cd^2+^ into yeast cells [65]. The authors concluded that LCT1 may contribute to the transport of the toxic Cd^2+^ across plant membranes.

Apart from potassium channels and transporters, the functional complementation of yeast mutants led to the characterisation of Na^+^-specific plant transport proteins. These include the tonoplast Na^+^/H^+^-antiporter AtNHX1 from the cells of *A. thaliana* [66]. The protein is homologous to the Nhx1 yeast transporter and complements the corresponding salt-sensitive *nhx1* mutant of *S. cerevisiae* [67,68]. Later, a family of *AtNHX1*-like genes of *A. thaliana* (*AtNHX1-5*), encoding for vacuolar-type Na^+^/H^+^ antiporters, was cloned and functionally characterised by their heterologous expression in yeast mutant *nhx1* [69]. The expression of all the *AtNHX* members of the family provided a recovery of the salt-sensitive yeast mutant, supporting their role in Na^+^/H^+^ exchange.

The functional complementation of the yeast *nhx1* mutant led to the characterisation of genes for NHX transporters from other plants, e.g., ThNHX1 protein from *Thellungiella halophila* (renamed as *Eutrema salsugineum*, a salt-tolerant relative of *A. thaliana*) [70], and TaNHX2 from wheat [71]. Both proteins have significant sequence homology with the NHX sodium exchanger in *Arabidopsis*. ThNHX1 presumably functions as a tonoplast Na^+^/H^+^ antiporter and plays an important role in the salt tolerance of *T. halophila* [70]. TaNHX2 encodes a K^+^/H^+^ antiporter of endomembranes; it is involved in cellular pH regulation and potassium nutrition under normal conditions [71].

The examples described in this section are far from providing a comprehensive list of studies with plant K^+^- and Na^+^-transporters that use yeast mutants. For a more detailed description, we recommend an extensive review by Locascio et al. [30], which is completely devoted to the subject.

### 2.3. Functional Complementation of S. cerevisiae Mutants to Study Other Plant Ion Channels

Heterologous expression in yeast mutants has been used to characterize the functions of some other plant channels. Examples of such studies include plant ion channels controlled by cyclic nucleotides (CNGCs, cyclic nucleotide-gated channels) [72]. Most plant CNGCs belong to non-selective cation channels residing in the plasma membrane [73]. They are formed by four subunits that are activated by cyclic nucleotide monophosphates, adenosine 3′,5′-cyclic monophosphate (cAMP) and guanosine 3′,5′-cyclic monophosphate (cGMP). CNGCs are mediators of numerous effects of cyclic nucleotides in a plant cell [74,75]. CNGCs are involved in diverse signalling pathways ranging from plant development to stress responses, including pathogen responses and heavy metal homeostasis. Twenty AtCNGC genes have been found in *A. thaliana* [76].

Yeast mutants deficient in K^+^ (*trk1 trk2*) or Ca^2+^ (*mid1 cch1*) uptake systems have been used for functional characterization of some plant CNGCs. The mutant *trk1 trk2* helped to characterize the genes *AtCNGC1*, *AtCNGC2* and *AtCNGC3* [77,78,79,80,81]. It was demonstrated that the proteins encoded by the genes partially (compared to the protein AtKAT1) complement the yeast mutant under low K^+^ concentrations.

An AtCNGC1 isoform was also functionally characterised in the yeast mutant *mid1 cch1* without two subunits of Ca^2+^ channel complex Cch1/Mid1 in the yeast plasma membrane, which mediates high-affinity Ca^2+^ influx and is involved in signal transduction in response to AMF (yeast alpha mating factor) [72]. The exposure of such a mutant to AMF leads to growth arrest. The full-length protein AtCNGC1 did not complement this mutant in response to the pheromone, whereas the protein with the deleted C-terminus restored the wild-type phenotype. These results led to the conclusion that AtCNGC1 is permeable to Ca^2+^ ions and that this permeability is regulated/inhibited by calmodulin interacting with the C-terminus of the protein [77].

Evidence has been presented supporting the existence of weak voltage-dependent nonselective cation channels (NSCC), which are the main pathways for Na^+^ entry into the roots at high soil NaCl concentrations [82,83]. CNGCs are among the subclasses of NSCCs that have been the subject of discussion in the context of Na^+^ fluxes [82,84]. Two CNGCs from *A. thaliana*, AtCNGC3 and AtCNGC10, have been linked to primary Na^+^ fluxes in the roots [75]. The Na^+^-transporting function of AtCNGC3 was studied by the expression in the salt-sensitive *S. cerevisiae* strain B31 (*ena1-4nha1*), which lacks genes of the main transport systems for Na^+^ export from the cytoplasm: the *ENA* cluster and *NHA1* [80]. The yeast mutant expressing AtCNGC3 was more sensitive to high salt concentrations; it also accumulated significantly more Na^+^ than the control cells transformed by the empty vector. The authors concluded that AtCNGC3 forms a functional Na^+^ permeable channel. The involvement of AtCNGC10 isoforms in Na^+^ transport was also demonstrated by the heterologous expression in yeast strain B31 [85].

The investigation of the mechanisms of absorption and regulation of Cl^−^ transport in plants is an important aspect of the study of plant salt resistance. Soil salinisation leads to the accumulation of not only Na^+^ but also Cl^−^ in the plant cell cytoplasm at toxic levels [86,87]. The proteins of the CLC family (**C**h**L**oride **C**hannel, family of anion channels and transporters) play an important role in the transport of Cl^−^ in prokaryotes and eukaryotes; members of this family participate in the transfer of not only Cl^−^ but also NO_3_^−^ [88]. In addition to chloride channels, this family includes anion/proton exchangers, Cl^−^/H^+^- and NO_3_^−^/H^+^-antiporters.

In plants, CLCs play key roles in anionic homeostasis, salinity tolerance and nitrogen nutrition (for reviews, see [89,90,91]). Plant CLC proteins are localised in endomembranes, where they perform many different functions: carrying out the electrogenic transport of NO_3_^−^ from the cytosol into vacuoles, regulating cytoplasmic concentrations of NO_3_^−^, participating in the acidification of organelle lumens and regulating their transmembrane electric potential. Seven genes of the CLC family, *AtCLCa–e*, have been cloned from *A. thaliana*, and the functions and physiological roles of their products have been intensively investigated [92].

To clarify the function of CLC genes, heterologous expression in the *S. cerevisiae* mutant strain *Δgef1* is used (along with other approaches such as the patch-clamp technique and expression in Xenopus oocytes). In *Δgef1*, the function of the only Cl^−^ channel/transporter, CLC homologue Gef1p, is distorted [93]. The cells with the disrupted *GEF1* gene fail to grow on Fe-deficient medium with nonfermentable carbon sources; also, the *Δgef1* mutant cannot grow under alkaline conditions and demonstrates hypersensitivity to some extracellular cations (Na^+^, Li^+^ and Mn^2+^) [93]. Heterologous expression of the Cl^−^ transporter in the *Δgef1* mutant restores the growth of the mutant on appropriate selective media. *Δgef1* has also been successfully used for clarifying the anion selectivity of CLC proteins in diverse organisms. For example, it was shown that AtCLCc, AtCLCd and AtCLCf from *A. thaliana* were able to complement the growth defect of the *Δgef1* yeast mutant, suggesting functional similarity with Gef1p [92,93,94]. The expression of CLC genes from rice (*Oryza sativa*), *OsCLC-1* and *OsCLC-2*, in the *Δgef1* mutant demonstrated that both genes encode chloride channels [95]. The same method was applied for the characterisation of Cl^−^ transporting CLC proteins from the moderately salt-tolerant *Glycine max* and the salt-tolerant wild species *Glycine soja*, namely, Cl^−^/H^+^ antiporter GmCLC1 and anionic channel GsCLCc2 [96,97].

Recently, the genes of anionic transporters of the CLC family from a salt-accumulating euhalophyte *Suaeda altissima* have been cloned [98,99,100,101]: *SaCLCa1/a2* (the putative orthologs of *AtCLCa* encoding NO_3_^−^/H^+^-antiporter of *A. thaliana* [102]), *SaCLCc1/c2* and *SaCLCd* (the putative orthologs, correspondingly, of *AtCLCc* and *AtCLCd* encoding Cl^−^/H^+^- antiporters of *A. thaliana* [103,104]), and *SaCLCf* and *SaCLCg* (the putative orthologs of *AtCLCf* and *AtCLCg* encoding Cl^−^ channels [94,105]). The CLC genes of *S. altissima* were expressed in the mutant strain *Δgef1*. This approach made it possible to characterise the functionality of the anionic carriers of *S. altissima*. The experiments demonstrated that two of the identified proteins, SaCLCa1 and SaCLCa2, are NO_3_^−^/H^+^-antiporters, and the other five proteins are Cl^−^ transport proteins: Cl^−^/H^+^-antiporters (SaCLCc1, SaCLCc2 and SaCLCd) or chloride channels (SaCLCf and SaCLCg).

### 2.4. Investigation of Plant Ion Pumps Using Mutants of Saccharomyces cerevisiae

One of the early publications on the expression of plant proteins in yeast was the original work on the expression of the plasma membrane (PM) H^+^-ATPase from *A. thaliana* in *S. cerevisiae* cells [106]. Yeast cells contain their own PM H^+^-ATPase with properties similar to those of the plant enzyme [107]. Knockout mutations in the PM H^+^-ATPase gene are lethal for yeast cells [108]. Therefore, to avoid the co-existence of yeast ATPase and recombinant ATPase in the same cells, the authors used the yeast strain in which the constitutive promoter of the yeast plasma membrane H^+^-ATPase gene *PMA1* was replaced by a galactose-dependent promoter *GAL1*. This strain expressing yeast ATPase Pma1, which could grow on galactose medium but not on glucose medium, was transformed with a plasmid carrying the coding region of plant H^+^-ATPase under the control of the yeast promoter *PMA1*. The promoter *PMA1* conferred a high level of constitutive expression of the heterologous protein. The resulting transformant strain expressed endogenous yeast ATPase and heterologous plant ATPase in a galactose medium, and plant ATPase was expressed in a glucose medium as well. In these experiments, the recombinant protein did not reach the plasma membrane and accumulated in an intracellular membrane system (ER); nevertheless, this research is the first to demonstrate that functional plant plasma membrane H^+^-ATPase can be synthesised in yeast cells in large amounts. In later experiments, using inducible promoters, which allowed independent control of the expression of the endogenous yeast H^+^-ATPase and that of the heterologous pump, three isoforms of the *Arabidopsis* pump, AHA1, AHA2 and AHA3, were expressed individually and their biochemical properties were characterised [109].

The moss *Physcomitrella patens* is salt-tolerant and able to grow at high concentrations of NaCl in a medium (up to 600 mM). This terrestrial plant has a gene encoding Na^+^-ATPase, a transport enzyme that is absent in higher vascular plants [20]. The function of the Na^+^-ATPase PpENA1 from *P. patens* was studied by heterologous expression in salt-sensitive *S. cerevisiae* strain B31 [20].

The functionality of some plant Ca^2+^-ATPases has been studied in yeast mutants with defective endogenous Ca^2+^ transport systems. In the yeast *S. cerevisiae*, Ca^2+^ homeostasis is maintained by P-type Ca^2+^-ATPases, Pmc1 (vacuolar Ca^2+^ pump), Pmr1 (Ca^2+^ pump of Golgi apparatus) and the vacuolar Ca^2+^/H^+^ antiporter Vcx1, which is inhibited by calcineurin and is not active at low Ca^2+^ concentrations [110]. In the yeast strain K616 (*pmr1 pmc1 cnb1*) [111], Pmc1, Pmr1 and the regulatory calcineurin subunit Cnb1, which is involved in controlling the activity of the yeast vacuolar Ca^2+^/H^+^ antiporter, are deleted, and the maintenance of Ca^2+^ homeostasis relies solely on Vcx1 activity. At physiological Ca^2+^ concentrations (≥1 mM Ca^2+^), the mutant grows as well as the WT, but growth is inhibited at low Ca^2+^ concentrations because of the inactivation of Vcx1, and complementation assays in the K616 strain are used for the functional analysis of Ca^2+^-ATPases [112]. Several types of IIA and IIB Ca^2+^-ATPases have been heterologously expressed in yeast mutants [16,113,114].

The function of the Ca^2+^-ATPase ECA1 from the endoplasmic reticulum of *A. thaliana* was characterised in yeast mutants defective in the Ca^2+^ pump of the Golgi apparatus (mutant *pmr1*) or in both the Ca^2+^ pumps of the Golgi apparatus and the vacuolar Ca^2+^ pump (mutant *pmr1 pmc1 cnb1*) [16].

In the K616 (*pmr1 pmc1 cnb1*) strain, auto-inhibited Ca^2+^-ATPases from *Arabidopsis*, isoform 2 (ACA2) and isoform 4 (ACA4), a calmodulin-regulated Ca^2+^-ATPase, were characterised [113,114]. Calmodulin-binding studies and complementation experiments demonstrated that the N-termini of ACA2 and ACA4 contain an auto-inhibitory domain with a binding site for calmodulin. ACA4, as well as Pmc1, the yeast vacuolar Ca^2+^-ATPase, conferred protection against osmotic stresses such as high NaCl, KCl and mannitol when expressed in the K616 strain. An N-terminally modified form of ACA4 specifically conferred increased NaCl tolerance, whereas full-length ATPase had less effect [114].

Yeast mutants defective in endogenous Ca^2+^ transport systems were used for expression and functional studies of the Ca^2+^-ATPase PpPCA1 from *P. patens* [115]. These studies demonstrated the presence of an autoinhibitory N-terminal domain in PpPCA1.

Vacuolar H^+^-pyrophosphatase functions in the tonoplast of plant cells to transport protons across the membrane at the expense of the energy released from the hydrolysis of inorganic pyrophosphate (PP_i_). Apart from the tonoplast, this enzyme is also found in the membranes of the Golgi apparatus. Thus, inorganic pyrophosphatase plays an important role in the regulation of vacuolar pH and also in the control of vesicular transport in plant cells [116]. Pyrophosphatase AVP1 from *A. thaliana* was expressed in the *vma1* mutant of *S. cerevisiae*, which was defective in the activity of V-type H^+^-ATPase and the H^+^-transporting activity of the studied heterologous enzyme [117]. Inorganic pyrophosphatase from *A. thaliana* complemented the transport function of yeast vacuolar H^+^-ATPase, thus confirming the ability of H^+^-pyrophosphatase to create a physiologically significant pH gradient at the vacuolar membrane.

### 2.5. Systems of Heterologous Expression and Research on Function of Mutant Protein Forms

Systems of heterologous expression help to elucidate the structure and functions of mutant protein forms. We illustrate what can be achieved using examples from the following studies.

Mutant forms of *Arabidopsis* KAT1, a hyperpolarisation-activated K^+^ channel expressed mainly in the stomatal guard cells, were investigated by a combination of random site-directed mutagenesis and screening of a *trk1 trk2* yeast strain transformed with the mutant KAT1 forms [118]. Strong modifications in the cation selectivity of the KAT1 mutant forms were revealed in this study. A sensitive site (T256) in the pore domain of the channel was found; mutations at this site significantly affected the cationic specificity of the channel.

High-affinity plant K^+^ transporters HAK together with inward rectifying K^+^ channels AKT1 carry the main contribution of ion uptake by roots; moreover, at low external K^+^ concentrations, the uptake of K^+^ is likely to occur in symport with H^+^ [119]. It was shown for the AtHAK5 transporter from *A. thaliana* that the mechanism is the only one for K^+^ uptake at extremely low (below 10 μM) external K^+^ concentrations [120,121]. The role of the transporter HAK5 is important under saline conditions [122]. Research by Aleman et al. [123] used heterologous expression of mutant HAK5 forms from *A. thaliana* in *S. cerevisiae* cells, allowing identification of the point mutation in *At*HAK5, which significantly increased the salt tolerance of yeast cells. The mutation at amino acid residue F130, which is highly conservative for HAK5 proteins from different species increased more than 100-fold the affinity of the transporter to K^+^ and decreased its affinity to Na^+^. The authors concluded that the amino acid residue F130 contributes to the formation of a selectivity filter of the *At*HAK5 transporter.

### 2.6. S. cerevisiae Serves as a Toolkit to Study Protein–Protein Interactions

Strains of baker’s yeast are used not only to identify the genes of ion transporters and ascertain the selectivity of the transporters in experiments with functional complementation of yeast mutants but also to understand protein–protein interactions. This important application of yeast mutants is aimed at finding the potential regulators that determine the activity of ion-transporting proteins.

Among the methods available for studying protein–protein interactions in vivo are Y2H (yeast two-hybrid), FRET (Förster resonance energy transfer), BiFC (bimolecular fluorescence complementation) and SLCA (split luciferase) (reviewed in [124]), but the techniques using heterologous expression in yeast cells remain, apparently, the most accessible and inexpensive. Already a classic approach to the detection of protein-protein interactions is the yeast two-hybrid system (Y2H, Yeast two Hybrid) [125,126]. Since its description in 1989 [127], this system has been widely used to identify protein–protein interactions in many organisms.

**Figure 4:**
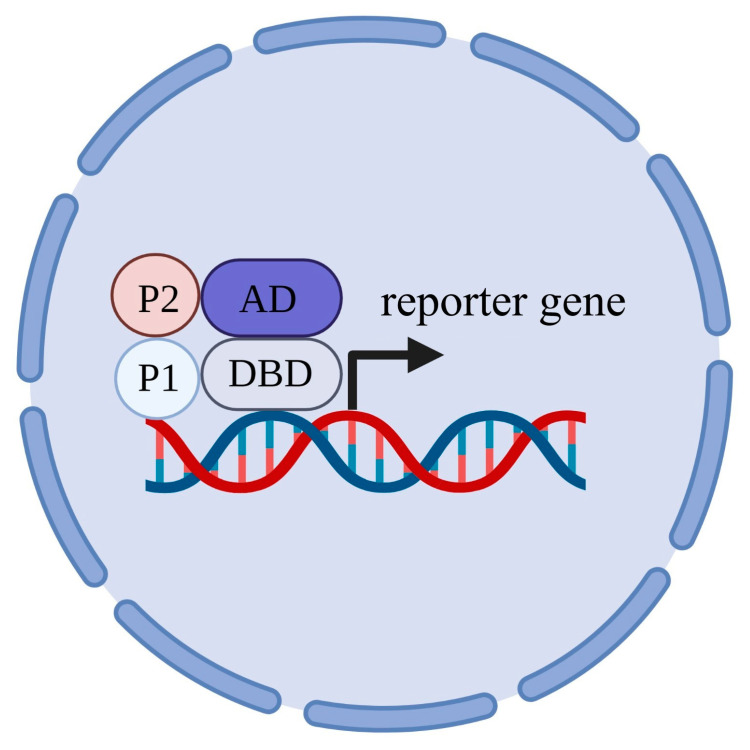
Scheme of classic yeast two-hybrid technique. DBD—DNA-binding domain; AD—activation domain; P1—“bait” (protein of interest, POI); P2—“prey”.

The heterologous expression of a single recombinant protein (protein of interest, POI) sometimes does not produce any distinct phenotypes under selective conditions. One of the reasons could be that it requires additional regulatory proteins, so the co-expression of the POI together with its regulators may solve this problem [124,125,126]. Y2H is based on the fact that many eukaryotic transcription factors include two functionally different domains, one of which ensures DNA binding and the other one controls the activation of transcription. For a classic operation of the technique [127], POI (so-called “bait”) is fused to the DNA binding domain of a transcription factor, which regulates the transcription of the reporter gene. The other protein under the study (“prey”) is fused to the activation domain, which is required to activate the transcription of the reporter gene by RNA-polymerase II. The physical interaction of the two studied proteins leads to the physical association of the DNA-binding domain with the activation domain of the transcription factor, restores its function and activates the transcription of the reporter gene (Figure 4). Genes *HIS3* and *ADE2* are often chosen as the reporter genes; these genes are required for the biosynthesis of histidine and adenine and are able to rescue the growth of histidine auxotrophic and adenine auxotrophic yeast strains, correspondingly, in media deficient in these compounds.

The accustomed way of using the yeast two-hybrid system has essential disadvantages [126]. For example, there is a high probability of false-positive or false-negative results, which could be a consequence of direct activation or inhibition of the heterologous proteins by themselves with the transcription of the reporter gene. Furthermore, the traditional yeast two-hybrid system is applicable for interactions between soluble proteins that could be transported to the nucleus; however, this system is not applicable for detecting protein–protein interactions for membrane proteins. At present, several modifications of the traditional yeast two-hybrid system have been developed that provide opportunities to overcome most of the abovementioned limitations of the system [128,129,130,131,132,133,134].

**Figure 5:**
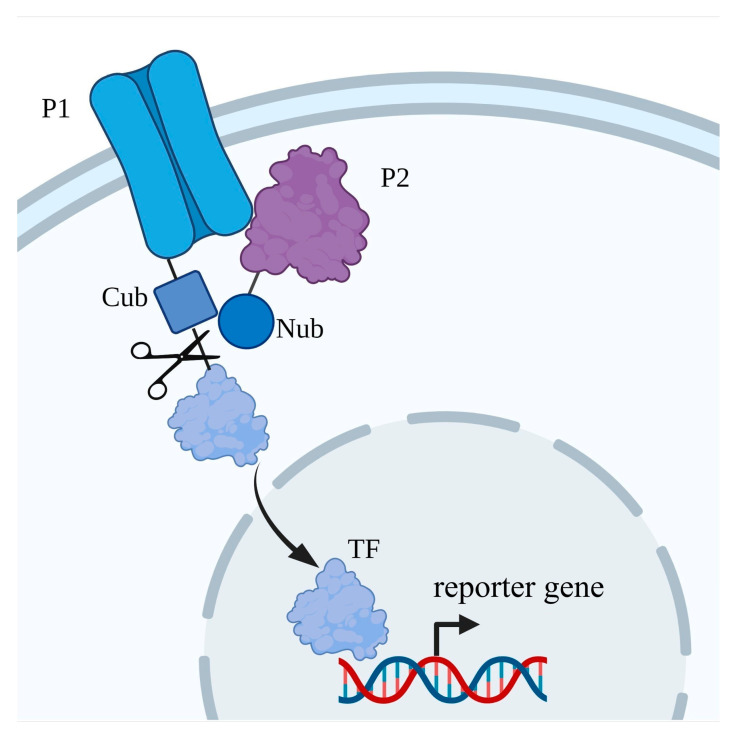
Scheme of split-ubiquitin Y2H system. P1—“bait” (protein of interest, POI), fused with C-end of ubiquitin (Cub); P2—“prey”, fused with N-end of ubiquitin (Nub); TF—transcription factor.

One of the modifications of the yeast two-hybrid analysis was adapted for the investigation of protein–protein interactions with the participation of membrane proteins. This method is based on the recovery of functional ubiquitin, the so-called split-ubiquitin Y2H system [126,131,132]. The protein used in this study is fused with the C-terminal fragment of ubiquitin and with the transcription factor that controls the expression of the reporter gene. The other protein used in this study is fused with the modified N-terminal fragment of ubiquitin with an amino acid substitution I13G, which renders this ubiquitin fragment low affinity to the C-terminal ubiquitin fragment (Figure 5). Since the transcription factor is linked by covalent bonds to the membrane protein and is localised outside of the nucleus, it is not able to activate the transcription from the reporter gene. However, when the two heterologous proteins in this study interact, the N- and C-terminal fragments of ubiquitin merge; the complete ubiquitin is recognised by the ubiquitin-specific proteases and finally the transcription factor is released. The transcription factor can then be transported to the nucleus and activate the transcription of the reporter gene (Figure 5).

Y2H as a powerful technique for the study of protein–protein interactions can be used to search for novel interacting partners by screening a single protein of interest or domain against a library of other proteins (i.e., libraries of genomic DNA, cDNA and ORF). The complete DNA library carries the prey element (prey library), and the POI is cloned into a plasmid as bait (bait plasmid). Commercial kits are available for performing such tasks, including the membrane-based yeast two-hybrid system, allowing the use of full-length integral membrane proteins and membrane-associated proteins as baits to hunt for unknown interaction partners. 

### 2.7. Protein–Protein Interaction with Ion Transporters of Plants That Were Discovered in S. cerevisiae

The abovementioned approaches were used to identify the proteins interacting with potassium and sodium transporters in plants (reviewed in [30]). For potassium transporters, the experiments revealed protein regulators for HKT transporters and shaker-type potassium channels. Different protein kinases, protein phosphatases, calcium sensors, GTPases, syntaxins and anion channels were identified among the proteins that influence the transport activity of potassium channels. Moreover, the phenomenon of homo- and hetero-oligomerisation was discovered for several transporters, such as AKT1/2, KAT1 (inward rectifiers), GORK and SKOR (voltage-gated outward rectifying K^+^-channels that allow K^+^ passage out of cells and open under membrane depolarisation (comprehensively reviewed in [135]).

The SOS system for the removal of excessive Na^+^ from plant cells is a good example where the expression of a single heterologous protein in yeast did not lead to a distinct phenotype under selective conditions but required additional components to activate the ion-transporting protein. *SOS* genes (*Salt Overly Sensitive*) were identified when screening mutants of *A. thaliana* with hypersensitivity to NaCl [136]. These genes were classified into three groups: *SOS1*, *SOS2* and *SOS3*. The genome of *A. thaliana* has one *SOS1* gene, 24 *SOS2* genes and 9 *SOS3* genes [137]. The SOS1 protein is a Na^+^/H^+^-antiporter in the plasma membrane, which is important for keeping low cytoplasmic Na^+^ concentrations. SOS1 proved to be one of the main determinants of salinity tolerance in plants [138]. SOS2 proteins are serine/threonine protein kinases that activate SOS1 by phosphorylating its C-terminal domain. In turn, the functioning of SOS2 proteins requires that they form a protein complex with the calcium-binding protein SOS3, which belongs to the family of calcineurin B-like proteins (CBLs). Therefore, the heterologous expression of SOS1 alone could not completely complement the salt-sensitive *S. cerevisiae* mutant B31 (*ena1-4 nha1*); the co-expression of *SOS1* with *SOS2* and *SOS3* essentially increased the salt tolerance of the B31 strain [139].

Several ion channels, including the inward-rectifying K^+^ channel KAT1, play a key role in the opening of stomata [140,141]. With KAT1 from *A. thaliana*, it was shown that this channel can interact with protein OST1 (open stomata 1) [142]. Protein OST1 (also known as SnRK2.6) is a stress-induced protein kinase that is involved in ABA signal transduction. The interaction between OST1 and KAT1 results in negative regulation of the ion channel (probably due to its phosphorylation), so the potassium fluxes into the guard cells decrease the stomatal aperture and water losses by the plant [142].

Among the many regulators of KAT1 is BCL2-associated athanogene4 (BAG4) (reviewed in [30]), which was revealed using the yeast split-ubiquitin system. BAG4 interacts with KAT1 and favours the trafficking of KAT1 to the plasma membrane. Two *Arabidopsis* mutant lines without the *BAG 4* gene exhibited a delay in the stomatal opening ([30] with references therein on several other regulators of KAT1).

A study of the model plant *A. thaliana* revealed a complex regulatory network in plant roots where the inward-rectifying potassium channel AKT1, which is responsible for potassium uptake from the medium, is gated (regulated) by Ca^2+^-dependent proteins. The interaction of calcineurin B-like protein (CBL, sensor of calcium) with CBL-interacting protein kinase 23 (CIPK23) led to the formation of an active protein complex that could bind to the C-terminal domain of the ion channel AKT1 [143]. Further phosphorylation of AKT1 by kinase CIPK23 activates the ion channel. Another participant of the regulatory network that interacts with AKT1 is a protein phosphatase of the 2C family (PP2C), AIP1 (AKT1-interacting PP2C1). The dephosphorylation of AKT1 by AIP1 inactivates K^+^ ion currents via AKT1. Deciphering the network for AKT1 regulation started with the aid of yeast two-hybrid systems and continued using *A. thaliana* mutants and other heterologous systems such as Xenopus oocytes for electrophysiological recordings [143,144]. Interestingly, transcripts of CBL and CIPK23 from *A. thaliana* restored the functions of the heterologously expressed AKT1 from barley [145], providing evidence for cross-species complementation in the system. Later development of the yeast two-hybrid system added more components (a silent K^+^ channel, KC1) to the AKT1 regulatory network [146].

## 3. Methylotrophic Yeast *Pichia pastoris* for Heterologous Expression of Proteins

Significant progress in structural studies of membrane proteins has been achieved using the methylotrophic yeast *Pichia pastoris* (at present, the yeast is reclassified to the genus *Komagataella*; the modern name is *Komagataella phaffi*) [11]. Although *S. cerevisiae* is considered a convenient model system for functional studies of heterologously expressed proteins, *P. pastoris* has the advantage of using yeast cells as factories for the production of large quantities of proteins of interest. *P. pastoris* accumulates a large biomass when cultured in inexpensive media with a simple composition and provides a high yield of heterologous proteins. The advantages of using methylotrophic yeasts include simple large-scale cultivation, the opportunity to use inexpensive methanol as a single source of carbon and a high yield of heterologously expressed protein. Yeasts are amenable to genetic manipulations [147,148]. Another peculiarity of methylotrophic yeasts is their capacity to realize post-translational modifications typical of proteins of higher eukaryotes [11]. There are numerous examples of how the system has been used for the expression of different membrane proteins, including GPCR [149], mammalian potassium channels [150], aquaporins [151] and transporters [152].

### 3.1. Vector Systems for Heterologous Expression in Cells of P. pastoris

**Figure 6:**
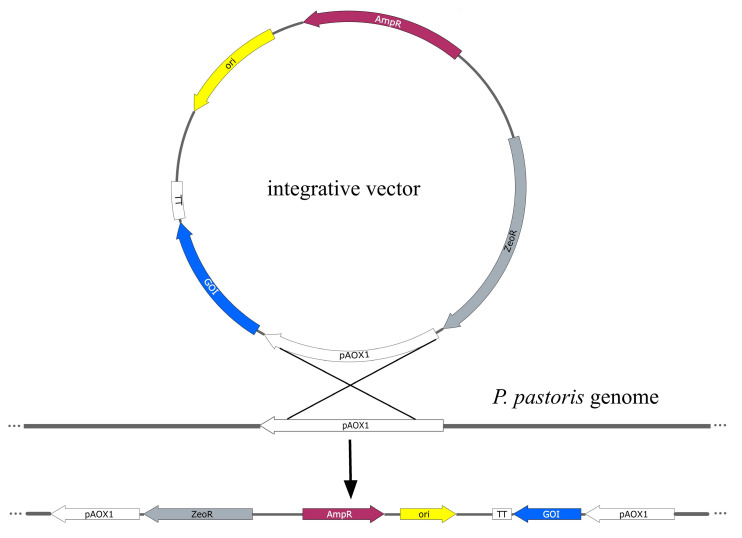
Scheme of recombinant integration of plasmid vector into *P. pastoris* genome. *pAOX1*—promoter of alcohol oxidase *AOX1* gene; AmpR and ZeoR—selective genes of ampicillin resistance and zeomycin resistance, respectively. TT—yeast terminator.

For *P. pastoris*, the vector constructs to express heterologous proteins are usually based on integrative vectors. After the transformation of *P. pastoris* cells, the construction integrates into the genome, providing clones that carry the target sequences [12]. This integration is ensured by the mechanism of homologous recombination and is realised at specific genomic loci (Figure 6). Typically for *P. pastoris*, it is the region of the promoter *pAOX1*; locus *pGAP* is also used (see later) [11]. The integration of the gene of interest into the yeast chromosome guarantees the stability of the resultant strains. Unfortunately, it is not possible to control the number of vector copies that integrate into the genome, so the optimal transformed clone has to be determined experimentally. A simple method is the screening of colonies in the presence of rising concentrations of the antibiotic zeocin [153]. Real-time PCR can be used to estimate the number of vector copies integrated into the genome [154].

Promoters are the key factors in producing strains, as promoters determine the gene expression level. Recombinant proteins in *P. pastoris* are usually expressed under the control of the strong constitutive promoter *pGAP* or under the control of the methanol-inducible promoter *pAOX1*. The cells of *P. pastoris* are able to utilize methanol as a single carbon source [11]. The first step in methanol utilisation involves the key enzyme, alcohol oxidase. The enzyme is localised in peroxisomes and catalyzes the oxidation of methanol to formaldehyde using molecular oxygen and forming hydrogen peroxide as a by-product [155]. Alcohol oxidase is represented by two isoforms encoded by the *AOX1* and *AOX2* genes. The methanol-inducible promoter *pAOX1* of the gene *AOX1* is often used in vectors for the heterologous expression of proteins in *P. pastoris* [11]. *pAOX1* is repressed when *P. pastoris* is grown on glucose, glycerol or ethanol [156]. The promoter is de-repressed when these carbon sources are exhausted, but it is completely induced by the addition of methanol alone. Separating the phases of growth and production allows biomass to accumulate until the phase of protein expression. As a consequence, yeast cells are not stressed by the accumulation of recombinant proteins during the growth phase. This makes it possible to produce proteins that are toxic to *P. pastoris*.

It is possible to use alternative constitutive promoters that do not need induction by methanol but ensure a high level of expression. Constitutive expression makes the process of production easier, excludes the use of potentially harmful inductors and ensures the steady transcription of the gene of interest. Usually, glyceraldehyde-3-phosphate dehydrogenase promoter *pGAP* is used; when grown on glucose, it helps to achieve nearly the same levels of recombinant protein expression as achieved under *pAOX1* when induced by methanol [157].

The development of a system in which the activation of *pAOX1* without methanol is possible [158,159,160]. For this system, the genes of the three repressors of *AOX1* were deleted, and a copy of the transcription factor MIT1 (methanol-induced transcription factor 1) was added under the control of the constitutive promoter *pGAP*. The resultant strain was able to express heterologous proteins under the control of *pAOX1* in a medium with glycerol (10 g/L) as the only carbon source [159]. The activation of the promoter *pAOX1* was achieved for the *P. pastoris* strain when glucose was substituted by glycerol. However, the repression of *pAOX1* in the growth phase for the strain required higher glucose than that for wild-type yeasts. Complete repression was observed under 40 g/L of glucose, and at 20 g/L of glucose, the reporter gene *GFP* could still be noticeably expressed [160].

At present, there are several strains of *P. pastoris* that are widely used for the expression of heterologous proteins [161]. The main feature of these strains is their ability to utilize methanol (Mut phenotype). Some strains such as GS115 have both functional genes of alcohol oxidase *AOX1* and *AOX2* (Mut^+^ phenotype). Other strains have the functionality of slowly expressing gene *AOX2* only, the strains grow slowly in media with methanol (Mut^S^ phenotype). An example of the latter is the strain KM71. There are also strains of *P. pastoris* with decreased proteolytic activity, such as SMD1168 (Mut^+^ protease deficient phenotype) [11].

### 3.2. The Factors That Influence Expression of Recombinant Proteins in P. pastoris

The membrane proteins in eukaryotic cells are synthesised in the membranes of the endoplasmic reticulum (ER). The translating ribosomes are directed to the membranes of the ER and are attached to the membranes following the signal recognition particle (SRP) pathway [162]. The components of the SRP pathway are the signal recognition particle (SPR) and its receptor (SR) located in the membrane of ER. Newly synthesised polypeptide chains cross the ER membrane during the translation with the aid of Sec61-translocon [163] and are folded with the aid of chaperones such as the Bip or PAT complex [164,165]. With the overexpression of membrane proteins, the availability of the abovementioned protein factors could be low and insufficient to ensure the correct folding and processing of the new polypeptide chains. In turn, this causes the accumulation of misfolded proteins, translation arrest and the activation of UPR (unfolded protein response) and ERAD (ER-associated protein degradation) systems [166,167].

The activation of UPR induces transcription of many genes whose products participate in the folding of proteins (chaperones) and biosynthesis of lipids [168]. The primary sensor that responds to the accumulation of misfolded proteins is the integral protein IRE1 of the ER membranes. The cytoplasmic domain of IRE1 has kinase and specific RNase activities [169]. If misfolded heterologous proteins do not accumulate in the ER, the sensor IRE1 is a monomer linked to the chaperone Bip. Abundant misfolded proteins in the ER govern the dissociation of the chaperone Bip from the monomers of IRE1, which allows the monomers of IRE1 to form oligomers. Within these oligomers, IRE1 is autophosphorylated and switches on its RNAse activity. The only substrate for the RNAse domain is the constitutively expressed mRNA of the transcription factor HAC1 [170]. The HAC1 mRNA contains an intron sequence that is not recognised by the spliceosome. The intron blocks the translation of HAC1, forming a specific secondary structure. Activated IRE1 performs splicing of mRNA for HAC1, removing the translational block [171]. Then, the synthesised protein HAC1 is transported to the nucleus and activates the transcription of genes responsible for UPR [171]. The complex mechanisms described for the folding of the expressed proteins and UPR are essential for the production of heterologously expressed proteins in *P. pastoris*. Overexpression of the active spliced form of HAC1 may either increase or decrease the yield of the heterologous protein in *P. pastoris,* depending on the nature of the protein [172].

Other factors influencing the yield of recombinant membrane proteins and the results of their subsequent crystallisation for structural studies include the frequency of nucleotides used in triplet codons, glycosylation of proteins and the presence of hydrophilic regions in the POI. The codons in the gene of POI can be optimised for successful heterologous expression in *P. pastoris*. For example, it was demonstrated that the optimisation of codon usage for the gene of murine P-glycoprotein (PgP) for its heterologous expression in *P. pastoris* resulted in a much higher (about 3-fold) yield of the heterologous protein compared to the wild-type gene of PgP [173]. Therefore, the substitution of codons that are rarely used by *P. pastoris* is more common for the yeast synonyms of nucleotide triplets, which may increase the production of heterologous proteins.

Yeast cells can glycosylate the proteins that enter their endoplasmic reticulum. Moreover, the pattern of glycosylation essentially differs between yeast cells and cells of higher eukaryotic organisms [11]. Both baker’s yeast *S. cerevisiae* and the cells of *P. pastoris* exhibit hypermannosylation of proteins, which is realised in the Golgi apparatus with the participation of the membrane protein α-1,6-mannosyltransferase Och1. However, the degree of glycosylation of proteins in *P. pastoris* is significantly lower than that in *S. cerevisiae*, mostly making up 10–20 mannose moieties in added oligosaccharide chains in *P. pastoris*, whereas, for *S. cerevisiae*, the number may reach 200 mannose units [174,175]. It is assumed that the removal of potential glycosylation sites benefits the formation of the protein crystals used to study the structure. At the same time, there are examples where 3D structures with high resolution are formed for the glycosylated forms of membrane proteins [176]. However, in most cases, excessive glycosylation leads to deviations from the correct protein folding and high heterogeneity in the structures of the recombinant protein. This is reflected in the functioning of the protein and its ability to crystallise. Several approaches have been developed to avoid the extra-glycosylation of heterologous proteins in *P. pastoris*. Deletion of the gene *OCH1* prevented hypermannosylation of heterologous proteins, but yeast mutants in *OCH1* exhibited abnormalities in the cell wall structure, with a higher tendency to lyse the cells and higher temperature sensitivity [177,178]. The other approach is to use a fungal enzyme, endoglycosidase endoT. The enzyme hydrolyses the bond between the moiety of N-acetylglucosamine, which is linked to the Asp of the protein and the remaining glycan chain. Therefore, the enzyme removes glycan chains from the expressed proteins (retaining only the first Asp-linked carbohydrate moiety), and the co-expression of endoT with the recombinant protein of interest leads to the expression of highly homogeneous proteins without glycosylation [179].

During the crystallisation stage, a new set of problems may arise for membrane proteins that contain large hydrophilic regions. To prevent difficulties, shorter truncated versions of the required protein are often expressed with deleted C- or N-terminal regions, and sometimes without hydrophilic loops between transmembrane domains. For example, deletion of the C-terminus was important for crystallisation of one of the GPCRs expressed in *P. pastoris* [149].

### 3.3. Approaches for Purification of Recombinant Proteins

The successful crystallisation of a protein for structural studies requires large amounts of well-purified protein material (milligrams or even tens of milligrams). Functional studies also require the proteins to be of high purity. An individual recombinant protein can be isolated by means of affinity chromatography and used further to study its function in the system of artificial proteoliposomes or crystallised to determine its 3D structure. For purification by affinity chromatography, the target protein synthesised in the yeast systems of heterologous expression carries an affinity tag at its N- or C-terminal ends. The tag can be a poly-His tag (for example, a His6-tag which binds to immobilised metal), glutathione S-transferase (GST, which binds to glutathione linked to the column matrix) or peptide FLAG (DYKDDDDK, which is used with a matrix carrying antibodies to FLAG) [180]. After the recombinant protein is bound to the affinity matrix and the other cellular proteins are washed out, the elution of this recombinant protein is carried out. High concentrations of imidazole (for proteins with His6 tag), free reduced glutathione (in case of GST tag) or free peptide FLAG is used for the elution [181].

**Figure 7:**
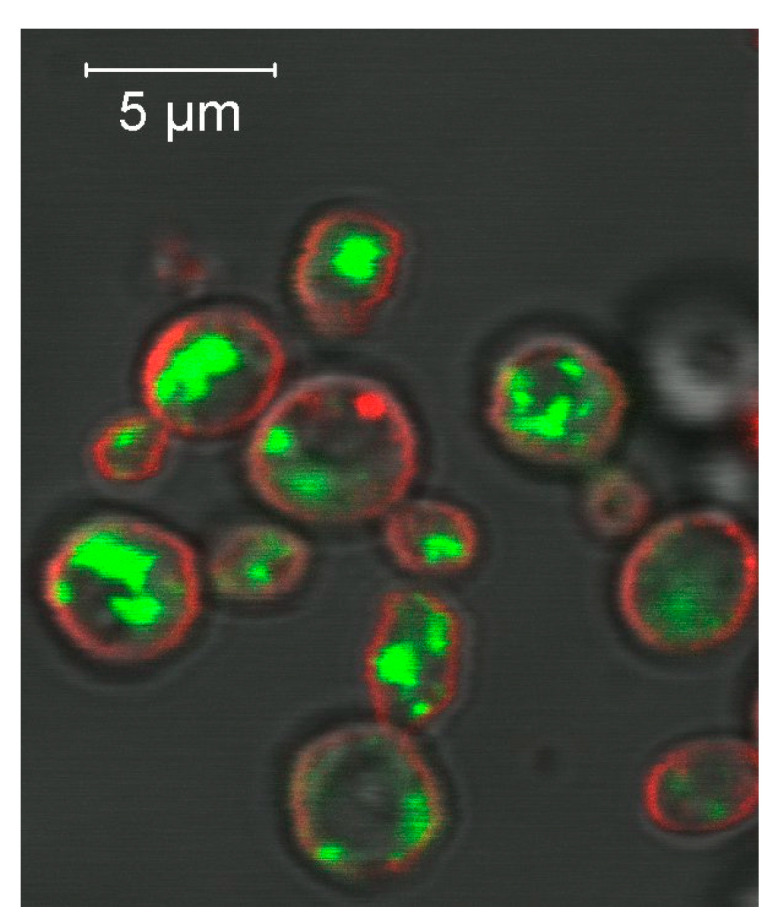
Gene (cDNA) of P-type ATPase from the marine microalga *Dunaliella maritima* similar to higher plant H^+^-ATPases (*DmHA2*; GenBank ID: KX 832225.1) fused with *GFP* coding sequence is expressed (green colour) in transformed *P. pastoris* cells (the GS115 strain). The plasma membranes of the yeast cells are stained with a fluorescent lipophilic dye FM 4-64 (molecular probes) (red colour).

Recombinant proteins can be fused with an indicator protein, e.g., with green fluorescent protein GFP, which helps to locate the fluorescent protein in a cell (Figure 7) and to trace the protein during its isolation and purification [180,182]. Before crystallisation, GFP and tag sequences have to be deleted. This can be achieved by introducing a specific site of recognition to the fused protein and cleavage by a protease such as TEV-protease or thrombin [180].

### 3.4. Heterologous Expression of Plant Proteins in Pichia pastoris

Despite the fact that the expression in *P. pastoris* is widely used for the study and production of proteins of bacterial, viral and animal origin, there are relatively few studies devoted to the study of plant proteins using this system of heterologous expression. *P. pastoris* has been mostly used for the expression of soluble plant proteins, such as barley soluble acid invertase [183], small plant GTPase RabA4c from *A. thaliana* [184], small heat shock proteins from *Camellia sinensis* [185], antifungal defensin 1 from *Pisum sativum* [186] and antifreeze protein from carrot [187] (for reference see [188]). Very few plant ion transporters have been expressed and functionally characterised in *P. pastoris*. Apparently, the study by Diatloff et al. [189] was the first successful attempt to express a plant ion transporter in *P. pastoris*. The authors carried out expression and functional characterisation of the low-affinity cation transporter (LCT1) from wheat (*Triticum aestivum*) in this organism. Formerly, when expressed in the yeast *S. cerevisiae*, LCT1 mediated the transmembrane transport of the monovalent cations (sodium, potassium, lithium, cesium and rubidium) [63] and the divalent cations calcium and cadmium [65]. Diatloff et al. [189] showed that LCT1 expressed in *P. pastoris* can transport Ca^2+^ and Cd^2+^. The transport characteristics of LCT1 expressed in *P. pastoris* confirmed LCT1 to be a low-affinity transporter [189].

Another example of the expression of plant membrane proteins in *P. pastoris* is the expression of aquaporin PM28A in spinach [190]. The protein (named SoPIP2;1 according to the new nomenclature [191]) is one of the major plasma membrane proteins in spinach leaves. Since plant species may have over 70 different aquaporins [192], the use of a heterologous expression system helps to avoid several similar isoforms in the protein preparations, as often happens when native membranes are used to isolate proteins of interest. In the study by Karlsson et al. [190], the overexpression of protein PM28A was realised in cells of *P. pastoris* under the *pAOX1* promoter; a high yield of 25 mg of purified protein from a liter of culture was reached. The protein was reconstituted in proteoliposomes and its function was studied. In another research, the aquaporin SoPIP2;1 was also produced with a high yield in the heterologous expression system in *P. pastoris* [193]. The overproduction of the spinach aquaporin in *P. pastoris* allowed the protein to be crystallized, its structure determined with a resolution of 2.1 Å, and details of its functioning at the molecular level were clarified [193].

Phytohormones play an important role in the growth and development of plants. In most cases, phytohormones are transported from the place of their synthesis to the place of their action. For some plant hormones and regulators, the process involves ABC transporters (ATP Binding Cassette transporters), particularly PDR transporters, which are responsible for pleiotropic drug resistance and are found only in plants and fungi [194,195]. The family of ABC transporters in plants is very large. For example, in *A. thaliana*, this family includes about 130 members [196]. As there are multiple forms of ABC transporters, it is necessary to have sufficient quantities of purified proteins for functional and structural investigations of an individual protein. However, plant PDR transporters are less studied than fungal transporters due to the lack of convenient systems for the heterologous expression of the plant transporters [197]. A previous study [197] describes the successful expression of *PDR* genes from *A. thaliana* in *P. pastoris*. The genes of the potential transporters of phytohormones, *PDR2* and *PDR8*, were cloned, fused with *GFP* and expressed; it was demonstrated that they reached their destination in the cell, the plasma membrane.

The SOS1 plasma membrane antiporter from *A. thaliana* has been expressed in *P. pastoris* for its structural and functional study [198]. The heterologous expression of two truncated forms of SOS1 was realised in the study. A slightly shorter form (from the 28th to the 990th amino acid residues) contained the predicted transmembrane domain and also 530 amino acid residues of the cytoplasmic C-terminal domain; a much shorter form was also expressed where nearly all the C-terminal domain was deleted. The expressed forms of the Na^+^/H^+^ antiporter were reconstructed in artificial proteoliposomes for functional studies. It was discovered that the shorter form of the protein with a nearly completely deleted C-terminal domain lost its activity to transport ions, whereas the form with a C-terminal domain kept its functional activity [198].

## 4. Conclusions

This review summarises a small part of the experimental data on the expression of plant membrane proteins, mainly various ion transporters, in yeast cells. The representative data are summed up in Table 1. The functional expression of genes of ion transporters in yeast heterologous hosts offers a remarkable opportunity to determine their transport activities. However, the use of yeast expression systems to study plant membrane proteins is not limited to this type of research work. A large number of publications have reported studies on other groups of plant transporters expressed in yeast cells, namely, sugar transporters, transporters of plant hormones, nucleotides and polyols; an extensive list is provided in other reviews, e.g., [9]. In general, the low cost of expressing heterologous proteins in yeast cells, together with the presence of major eukaryotic cellular mechanisms of protein processing in yeasts, provide opportunities to produce large amounts of purified recombinant proteins and make the yeast systems attractive experimental tools to produce preparative amounts of protein necessary for further functional and structural studies.

## Figures and Tables

**Table 1:** Some plant proteins whose function has been determined by heterologous expression in yeast *S. cerevisiae* and *P. pastoris*.

Protein Expressed	Source Species	Protein Function	Reference, Yeast Species
AtPT1, AtPT2	*Arabidopsis thaliana*	Phosphate transporters	[15], *S.c.*
ECA1	“	Ca^2+^-ATPase of ER	[16], *S.c.*
NRT1.1	“	Nitrate transporter	[17], *S.c.*
AtPTR1	“	Peptide transporter of PM	[18], *S.c.*
LeST1-1; LeST1-2	*Lycopersicon esculentum*	Sulphate transporters	[19], *S.c.*
KAT1	*A. thaliana*	PM K^+^ inward rectifying channel (guard cells)	[46,142], *S.c.*
OsKAT1	*Oriza sativa*	PM K^+^ inward rectifying channel (guard cells)	[49], *S.c.*
AKT1	*A. thaliana*	PM K^+^ inward rectifying channel (root cells)	[47], *S.c.*;
TaHKT2;1 (HKT1)	*Triticum aestivum*	K^+^/Na^+^-symport (subfamily II of HKT family)	[59,60], *S.c.*
AtHKT1;1	*A. thaliana*	Na^+^ transport (subfamily I of HKT family)	[61], *S.c.*
SeHKT1;2	*Salicornia europaea*	Na^+^ transport (subfamily I of HKT family)	[62], *S.c.*
LCT1	*Triticum aestivum*	Low affinity cation transporter; low affinity Na^+^ and K^+^ uptake; high affinity Ca^2+^ and Cd^2+^ uptake	[63], *S.c.* [189], *P.p.*
AtNHX1	*A. thaliana*	Tonoplast Na^+^/H^+^ antiporter	[67], *S.c.*; [68], *S.c.*;
ThNHX1	*Thellungiella halophila*	Tonoplast Na^+^/H^+^ antiporter	[70], *S.c.*
TaNHX2	*Triticum aestivum*	K^+^/H^+^ antiporter of endomembranes	[71], *S.c.*
AtCNGC1	*A. thaliana*	Cyclic nucleotide gated channels, permeable to K^+^, calmodulin regulated (putative Ca^2+^ permeability)	[78], *S.c.*
AtCNGC2	*A. thaliana*	Cyclic nucleotide-gated channel, permeable to K^+^	[78], *S.c.*
AtCNGC3	*A. thaliana*	Cyclic nucleotide-gated channel, permeable to Na^+^, K^+^	[80], *S.c.*
AtCNGC10	“	Cyclic nucleotide-gated channel, permeable to Na^+^	[85], *S.c.*
AHA1 AHA2 AHA3		H^+^-ATPase of PM	[106] *S.c.* [109], *S.c.*
PpPCA1	*Physcomitrella patens*	PIIB-type Ca^2+^-ATPase	[115], *S.c.*
PpENA1	*Physcomitrella patens*	Na^+^-ATPase	[20], *S.c.*
AVP1	*A. thaliana*	Vacuolar H^+^-Pyrophosphatase	[117], *S.c.*
AtCLCa	“	NO_3_^−^/H^+^ antiporter (vacuolar membrane)	[92,102], *S.c.*
AtCLCb	“	NO_3_^−^/H^+^ antiporter (vacuolar membrane)	[92], *S.c.*
AtCLCc	“	vacuolar Cl^−^ transporter (probably, Cl^−^/H^+^ antiporter)	[92,103], *S.c.*
AtCLCd	“	Cl^−^/H^+^ antiporter (trans-Golgi network)	[95], *S.c.*
AtCLCe	“	Cl^−^ channel of thylakoid membranes	[94], *S.c.*
AtCLCf	“	Cl^−^ channel of Golgi membranes	[94], *S.c.*
AtCLCg	“	Cl^−^ channel (vacuolar membrane)	[92], *S.c*
OsCLC-1; OsCLC-2	*O. sativa*	Cl^−^ channels (vacuolar putative)	[95], *S.c.*
GmCLC1	*Glycine max*	Cl^−^/H^+^ antiporter	[96], *S.c.*
GsCLC-c2	*Glycine soja*	Anionic (Cl^−^ and NO_3_^−^) channel	[97], *S.c.*
SaCLCa1/a2	*Suaeda altissima*	NO_3_^−^/H^+^ antiporter	[99,101], *S.c.*
SaCLCc1/c2	*S. altissima*	Cl^−^/H^+^ antiporter	[98,101], *S.c.*
SaCLCd	*S. altissima*	Cl^−^/H^+^ antiporter	[100], *S.c.*
SaCLCf; SaCLCg	*S. altissima*	Cl^−^ channels, putative	[100], *S.c.*
AtHAK5	*A. thaliana*	High-affinity K^+^-transporter of PM	[123], *S.c.*
SOS1	*A. thaliana*	Na^+^/H^+^ antiporter of PM	[139], *S.c.*; [198], *P.p.* [190], *P.p.*
SoPIP2;1 (PM28A)	*Spinacia oleracea*	Aquaporin of PM	[193], *P.p.*;
PDR2; PDR8	*A. thaliana*	Potential transporters of phytohormones in PM (ABC family transporter)	[197], *P.p.*

## Data Availability

Data are contained within the manuscript.
